# Payment for multiple forest benefits alters the effect of tree disease on optimal forest rotation length

**DOI:** 10.1016/j.ecolecon.2017.01.008

**Published:** 2017-04

**Authors:** Morag F. Macpherson, Adam Kleczkowski, John R. Healey, Nick Hanley

**Affiliations:** a Computing Science and Mathematics, School of Natural Sciences, University of Stirling, Cottrell Building, Stirling FK9 4LA, UK; b School of Environment, Natural Resources and Geography, College of Natural Sciences, Bangor University, Bangor, Gwynedd LL57 2UW, UK; c School of Geography & Geosciences, University of St Andrews, Irvine Building, North Street, St Andrews, Fife KY16 9AL, UK

**Keywords:** Payment for ecosystem services, Payment for environmental services, Forest ecosystem services, Green payments, Invasive species, Pests and diseases, Hartman model, Bioeconomic modelling, Optimal rotation length

## Abstract

Forests deliver multiple benefits both to their owners and to wider society. However, a wave of forest pests and pathogens is threatening this worldwide. In this paper we examine the effect of disease on the optimal rotation length of a single-aged, single rotation forest when a payment for non-timber benefits, which is offered to private forest owners to partly internalise the social values of forest management, is included. Using a generalisable bioeconomic framework we show how this payment counteracts the negative economic effect of disease by increasing the optimal rotation length, and under some restrictive conditions, even makes it optimal to never harvest the forest. The analysis shows a range of complex interactions between factors including the rate of spread of infection and the impact of disease on the value of harvested timber and non-timber benefits. A key result is that the effect of disease on the optimal rotation length is dependent on whether the disease affects the timber benefit only compared to when it affects both timber and non-timber benefits. Our framework can be extended to incorporate multiple ecosystem services delivered by forests and details of how disease can affect their production, thus facilitating a wide range of applications.

## Highlights

• A bioeconomic model of a multiple output forest with disease is proposed.
• The optimal rotation length is found for a range of key parameters.
• A payment for non-timber benefits counteracts the negative effect of disease.
• Structure of the payment, if it is dependent on the level of disease, is critical.

## 1 Introduction

Forests supply a wide range of important ecosystem services such as the regulation of hydrological and carbon cycles (Carvalho-Santos et al., 2014, Cudlín et al., 2013); recreational and aesthetic values (Nielsen et al., 2007, Ribe, 1989); as well as the conservation of biodiversity (Johansson et al., 2013). They can also provide timber revenues to private forest owners and managers. However, like many other natural resources, forests are experiencing many challenges, one of which is the increasing pressure from novel pests and pathogens (Gilligan et al., 2013). Changing climate (Netherer and Schopf, 2010, Pautasso et al., 2010, Sturrock, 2012), globalisation of trade and the synonymous increase in the volume and diversity of plant species and products being traded (Gilligan et al., 2013, Work et al., 2005) are just a few of the causes of an increase in geographical ranges of pest and pathogen species. With these factors unlikely to diminish in the near future, it is very important to consider the effect of disease on multiple-output forests and how they are managed. More specifically, in this paper we consider the management decision of the time of clear-felling and ask: what is the effect of disease on the optimal rotation length of a multiple-benefit forest?

How to modify forest management to make forests less susceptible to climate change effects has become a popular theme in the literature (Millar et al., 2007), and while climate and disease risks are intricately linked (Loehle et al., 2016, Sturrock et al., 2011), there appears to be far less material on the adaptation of forest management to create greater protection against tree diseases. Some strategies that are reported in the literature are tree species diversification (Castagneyrol et al., 2014, Churchill et al., 2013, Jactel and Brockerhoff, 2007, Perry and Maghembe, 1989), alteration of spatial structure (Condeso and Meentemeyer, 2007) and adapting silvicultural practices such as thinning (Bauce and Fuentealba, 2013, D’Amato et al., 2011). More recently, Marzano et al. (2017) identified 33 disease management options applicable to combat the needle blight pathogen of *Pinus* spp. trees *Dothistroma septosporum*, ranging from increasing knowledge of the pathogen system to changes in initial forest design, such as lower initial tree stocking density. Most of these strategies are preventative and attempt to reduce the risk of initial infection. This is largely because there is little that can be done to combat most pathogens once they have arrived. However, some within-rotation options include: a heavier thinning regime (for example against *D. septosporum*; Quine et al. (in preparation)); chemical sprays or biological control (for example treating stumps with urea or a biological control agent *Phlebiopsis gigantea* can help prevent germination and growth of aerial basidiospores of *Heterobasidion annosum* that causes root and butt rot of conifers; (Johansson et al., 2002) ; and clear-felling the forest early (for example in the case of widespread epidemics). All these management strategies and decisions have direct implications not only for timber production but also for the non-timber services that are produced by forests. For example in 2013–14, 575 sites in the UK were served with a Statutory Plant Health Notice requiring a total of 4.8 thousand hectares of forest to be felled in a bid to halt the progression of the pathogen *Phytophthora ramorum* (Forestry Commission Scotland, 2015). Such removal of timber not only affects the forest owner through revenue loss, but may also negatively affect the supply of non-timber benefits, e.g. through habitat loss which may disrupt wildlife (Appiah et al., 2004, Rizzo and Garbelotto, 2003). Thus, management decisions should anticipate the effect of pests and diseases on both the timber and the non-timber benefits of a forest. This is the focus of our paper.

Finding the optimal rotation length for a forest when disease is present is an economically important decision for a forest manager, since the arrival of pests and pathogens can lead to losses in market values through: reduction in tree growth, for example *D. septosporum* causes significant defoliation that can greatly reduce growth rate (Mullett, 2014); reduction in timber quality of live trees, for example *Heterobasidion annosum* decays the wood in the butt end of the log which may reduce the value of the timber (Pratt, 2001, Redfern et al., 2010); an increase in the susceptibility to secondary infection, for example *Hymenoscyphus fraxineus* and *Phytophthora ramorum* causes significant damage to the bark and cambium therefore increasing the rate of infection of wood decay fungi (Scotland, 2015, Pautasso et al., 2013); or at the scale of the forest stand the disease may increase the proportion of trees that are dead and thus subject to wood decay, for example *Ips typographus* has killed trees in more than 9000  ha of *Picea abies* forest in Europe. In the case of an epidemic, large areas of monoculture forest may be felled simultaneously to try to halt disease spread (as is currently taking place in response to the *P. ramorum* infection of *Larix* spp. in South Wales and South West Scotland (Forestry Commission Scotland, 2015), thus a large influx of material to local sawmills may cause congestion and market saturation (however we do not model this scenario explicitly as that would require a reduced price for all timber independent of its infection status).

Despite the important impact of tree pests and pathogens, and the variety of analyses within the optimal rotation length literature (Newman (2002) found 313 published books and articles in over sixty journals since Faustmann's novel paper on optimal rotation length analysis), there is a lack of published work linking the effect of disease to the optimal rotation length. In Macpherson et al. (2016) we analyse the effect of disease on the optimal rotation length of an even-aged forest by creating a generalisable, bioeconomic model framework, which combines an epidemiological, compartmental model with a single-rotation Faustmann model (describing the net present value, NPV, of a forest by including a one-off establishment cost and timber revenue; (Amacher et al., 2009) ). We found a key trade-off between waiting for the timber to grow and the further spread of infection over time: the optimal rotation length, which maximises the NPV of the forest, is reduced when timber from infected trees has no value, but when the infection spreads quickly, and the value of timber from infected trees is non-zero, it can be optimal to wait until the disease-free optimal rotation length to harvest. However, this set-up is representative of plantation forests where management decisions are driven by timber production only (and non-timber values are not considered).

It is, however, commonly recognised that the value of forests extends beyond timber; and Faustmann's original model has since been extended to include the benefits of non-timber goods (Hartman, 1976, Samuelson, 1976). Hartman (1976) showed that ignoring such benefits can lead to a suboptimal rotation length. Since then, the inclusion of non-timber benefits has become a cornerstone of optimal rotation length analysis, with studies examining the effect of including: the cost of maintaining the provision of recreational services (Snyder and Bhattacharyya, 1990); carbon sequestration, taxes or subsidies (Englin and Callaway, 1993, Price and Willis, 2011, Van Kooten et al., 1995); timber and carbon sequestration benefits while maintaining a given level of biodiversity in a single forest (Nghiem, 2014); and the interdependence of the provision of amenity services from adjacent forests (Koskela and Ollikainen, 2001, Swallow and Wear, 1993). These models generally depend on a function that describes the production of timber and non-timber benefits through time. It is (relatively) easy to quantify the timber value of a forest using appropriate species yield growth curves, and the timber price can be taken from market data. It is harder to do this for non-market benefits; however, recent techniques for valuing non-timber benefits have been developed (such as contingent valuation), and this can help inform the functions describing the non-timber benefits in such models (Bishop, 1999).

In this study we extend the bioeconomic model in Macpherson et al. (2016) by assuming that the forest owner has an interest in non-timber benefits such as biodiversity, carbon sequestration and/or recreation as well as timber benefits priced by the market. We do this by including a “green” payment which provides an economic incentive for the private forest owner to take into account the non-timber benefits of retaining tree cover when making decisions (the NPV of the forest is therefore similar to a single-rotation, Hartman model). This green payment could be thought of as a form of payment for ecosystem services; and we assume that it increases linearly dependent on the area of the forest. While a simplification, this allows us to investigate the effect of disease on the optimal rotation length of a multiple-output forest and undertake analysis of sensitivity to key parameters (describing the spread of infection and impact of disease on the timber and non-timber values); we also discuss how the function describing the non-timber benefits can be adapted to depend on other forest attributes (such as the age of the trees) in the Discussion section.

Traditional optimal rotation length analysis is conducted over multiple rotations where trees are perpetually planted and harvested, thus synonymously incorporating the benefit of the land (Amacher et al., 2009). In our model we analyse the effect of disease on the optimal rotation length over a single rotation, and use a ‘land rent’ term to include the future benefit after harvest. Including multiple rotations in our model in a more specific way would require an assumption of what happens to the level of infection between rotations (i.e. if and how the pathogen carries over to the next rotation after a harvest). This adds much complexity to the system since the carry-over of disease is very pathogen specific. Moreover, despite the use of multiple rotations to find the optimal rotation length in modelling the effects of other catastrophic events (such as fire or wind; (Englin et al., 2000) ), these disturbance events have many dissimilarities with disease. These include: the speed of progression, the symptoms, the management response once detected, the potential to salvage timber and the irreversibility due to long-term persistence of many pathogens following their invasion. Therefore, we use a single rotation set-up with land rent after harvest in order to focus on the central issue of our paper: the interaction of disease with timber and the non-timber benefits.

The first key aim of this paper is to use the bioeconomic model to examine what effect disease has on the optimal rotation length of a multiple-output forest. We recognise, however, that disease can affect the provision of non-timber outputs differently. For example, a disease that reduces the growth rate of trees, such as *D. septosporum* on *Pinus* spp., may decrease the timber revenue but have a limited impact on non-timber benefits such as biodiversity and recreation (however the rate of carbon sequestration associated with tree growth may also be affected; (Hicke et al., 2012) ). Alternatively, pathogens like *Ophiostoma ulmi* and *O. novo-ulmi* on *Ulmus* spp. or *Cryphonectria parasitica* on *Castanea dentata*, cause widespread tree mortality reducing both timber and non-timber benefits such as the loss of biodiversity, carbon storage, and recreation and aesthetic values (Boyd et al., 2013, Gilligan et al., 2013, Hicke et al., 2012). A second aim of this paper is therefore to consider how the formulation of the green payment affects the optimal rotation length. We do this by considering two green payment functions: the first assumes that disease affects the timber benefit only (and thus the non-timber benefits remain unaffected), and the second assumes that disease affects both the timber and non-timber benefits. This analysis provides an exemplar framework that could be adapted for a specific host-pathogen systems with specific forest (timber and non-timber) benefits.

The structure of this paper is as follows. In Section 2 we find the first-order condition for a single rotation, Hartman model and then extend the framework to include a general disease system. In Section 3 we introduce a specific timber volume function and susceptible-infected (SI) compartmental model. We use this in the general model to highlight some key results produced by numerical optimisation for two cases (first when disease affects the timber benefits only, and the second when disease affects both the timber and non-timber benefits) in Section 4, and then close with a summary and discussion in Section 5.

## 2 Formulation of the General Model

### 2.1 The Model without Disease

We develop a single rotation Hartman model, where the NPV of an even-aged, monoculture forest includes the establishment cost (planting from bare land), the benefit from harvesting the timber, a non-timber green payment (Hartman, 1976), and a land rent payment *after* the forest rotation. While the objective function is similar to that of Hartman (1976), we first explain our formulation without disease so that it is easy to understand how we then incorporate the effect of disease on each term (in Section 2.2).

We assume that for a forest of area *L* (in hectares), the establishment costs are linearly dependent on the area, *W*(*L*) = *cL* where *c* is the planting cost per hectare. The net benefit of harvesting, *M*(*L*,*T*), is a product of the per-cubic-metre price of timber, *p*, and the volume of timber produced, *f*(*T*)*L*. The annual green payment is linearly dependent on the area of the forest, *S*(*L*) = *sL* where *s* is the payment per hectare per year and is obtained for as long as the trees remain unharvested. We also include an annual payment for land rent *after* harvesting that is linearly dependent on the area, *A*(*L*) = *aL* where *a* is the payment per hectare per year obtained after the trees are harvested. Further underlying assumptions include: all costs and prices are constant and known; future interest rates are constant and known; and the timber volume function of the species is known (Amacher et al., 2009). Thus the NPV of a forest with a rotation length of *T* years is

(1) $$Ĵ(T)=-W(L)+M(L,T)e^{-rT}+\int_{0}^{T}\;S(L)e^{-rt}\;dt+\int_{T}^{\infty }\;A(L)e^{-rt}\;dt.$$

An exponential discount factor, with rate *r*, is used to discount future revenue (from the timber harvest, green payment and land rent) back to the time of planting. Undertaking the integrations in Eq. (1) and substituting the function for the revenue from harvesting we obtain

(2) $$Ĵ(T)=-W(L)+pf(T)Le^{-rT}-\frac{S(L)}{r}\left(e^{-rT}-1\right)-\frac{A(L)}{r}\left(-e^{-rT}\right).$$

Parameter definitions and baseline values are given in Table 1. To find the optimal rotation length which maximises the NPV, we find the first-order condition by differentiating Eq. (2) with respect to *T* which gives

(3) $$\frac{dĴ(T)}{dT}=p\frac{df}{dT}Le^{-rT}-rpf(T)Le^{-rT}+S(L)e^{-rT}-A(L)e^{-rT}.$$

Setting Eq. (3) equal to zero we obtain

(4) $${\left(\frac{1}{f(T_{DF})}\frac{df}{dT}\right|}_{T=T_{DF}}-r=\frac{A(L)-S(L)}{pf(T_{DF})L}.$$

This implies that the optimal rotation length for the disease-free system (*T* = *T*_*DF*_), which maximises the NPV in Eq. (1), is given when the value of marginal gain from the relative growth in timber volume and the opportunity cost of investment (left-hand side) is equal to the future land rent minus the non-timber benefits relative to the timber revenue (right-hand side). The green payment is designed to increase the benefit of retaining the cover of the current tree crop for longer, and Eq. (4) shows this since an increase in the green payment will increase the benefit obtained from delaying the harvest, and therefore increase the optimal rotation length.

Evaluating the second derivative at the optimal rotation length gives

(5) $${\left(\frac{d^{2}Ĵ}{dT^{2}}\right|}_{T=T_{DF}}=pLe^{-rT_{DF}}\left({\left(\frac{d^{2}f}{dT^{2}}\right|}_{T=T_{DF}}-r{\left(\frac{df}{dT}\right|}_{T=T_{DF}}\right)<0,$$

which holds if the timber volume has an increasing, concave function.

### 2.2 General Model with Disease

We now examine the effect of disease on the optimal rotation length by incorporating two parameters which scale the revenue obtained from the timber and non-timber benefits of infected trees. We first introduce the NPV and the general disease system, and finally derive the first-order condition which allows us to show the effect of disease on the optimal rotation length.

Eq. (1) represents the NPV for a forest of area *L* which remains disease free. We build on this model, by assuming that the revenue obtained from the timber and the green payment is dependent on the state of infection at that point in time. Therefore the NPV can be given by

(6) $$Ĵ(T)=-W(L)+M({\tilde{L}}_{TB}(T),T)e^{-rT}+\int_{0}^{T}S({\tilde{L}}_{NTB}(t))e^{-rt}\;dt+\int_{T}^{\infty }A(L)e^{-rt}\;dt$$

where ${\tilde{L}}_{TB}(T)$ and ${\tilde{L}}_{NTB}(T)$ denote the effective area of forest providing timber and non-timber benefits in the presence of disease respectively (explained further below). The establishment cost and the land rent remain unchanged.

Next we assume that, for a general pathogen a tree can be in one of *N* states of infection. We denote the area of the forest in the *i*th state by *x*_*i*_(*T*) at the time of felling, where 1 ≤ *i* ≤ *N*. Since no partial felling is undertaken the total land area under tree cover is unchanged, giving the condition $L=\sum_{i=1}^{N}x_{i}(T)$. First consider the effect of disease on the timber benefit. If the disease had no effect on timber value, the revenue from timber in the *i*th state of infection is *pf*(*T*)*x*_*i*_(*T*). However, we assume that the disease reduces the value of timber (either through reduced quality or growth), so the revenue from timber in each state is scaled by parameter *ρ*_*i*_ where 0 ≤ *ρ*_*i*_ ≤ 1. This means that timber may be affected differently by disease between the states. We can therefore represent the revenue from harvested timber as

(7a) $$M\left(\tilde{L}(T),T\right)=pf(T)\left(\overset{N}{\underset{i=1}{\sum }}\rho_{i}x_{i}(T)\right)$$

(7b) $$=pf(T){\tilde{L}}_{TB}(T)$$

where the effective area of the forest providing a timber benefit in the presence of disease at time *T* is given by

(8) $${\tilde{L}}_{TB}(T)=\overset{N}{\underset{i=1}{\sum }}\rho_{i}x_{i}(T).$$

We assume $d{\tilde{L}}_{TB}(T)/dT\leq 0$ since it is usual that the damage caused to the timber by disease has a permanent negative effect.

Similarly, we assume that the green payment for the area of trees in the *i*th state of infection is scaled by parameter *σ*_*i*_ to represent the effect of disease on the non-timber benefits, where 0 ≤ *σ*_*i*_ ≤ 1. Thus the annual green payment term in Eq. (6) is

(9a) $$S\left({\tilde{L}}_{NTB}(T)\right)=s\left(\overset{N}{\underset{i=1}{\sum }}\sigma_{i}x_{i}(T)\right)$$

(9b) $$=s{\tilde{L}}_{NTB}(T)$$

where the effective area of the forest providing non-timber benefits in the presence of disease at time *T* is given by

(10) $${\tilde{L}}_{NTB}(T)=\overset{N}{\underset{i=1}{\sum }}\sigma_{i}x_{i}(T).$$

Unlike the effect of disease on the timber benefit, we make no assumption that ${\tilde{L}}_{NTB}(T)$ is an increasing or decreasing function since it is not needed for the results that we show. (Note, however, that the effect of disease on the non-timber benefits would be likely to depend on the specific non-timber ecosystem service being modelled.)

The spread of infection throughout the forest is included in this model framework by specifying a system of differential equations (*dx*_*i*_/*dT*) that can be solved for *x*_*i*_(*T*), and substituted into the harvest revenue function (Eq. (7b)) and green payment function (Eq. (9b)). To find a general solution we differentiate Eq. (6) with respect to *T*, which gives

(11) $$\begin{array}{ll} \frac{dĴ(T)}{dT}= & pe^{-rT}\left(\frac{df}{dT}{\tilde{L}}_{TB}(T)+f(T)\frac{d{\tilde{L}}_{TB}}{dT}-rf(T){\tilde{L}}_{TB}(T)\right)\\ & +\frac{d}{dT}\left(\overset{\;T}{\underset{\;0}{\int }}\;S\left({\tilde{L}}_{NTB}(t)\right)e^{-rt}\;dt\right)-A(L)e^{-rT} \end{array}$$

Setting Eq. (10) equal to zero and re-arranging we obtain the first-order condition,

(12) $$\begin{array}{c} \left(\frac{1}{f(T_{D})}\frac{df(T)}{dT}\right|T=T_{D}-r\\ \;=\frac{1}{{\tilde{L}}_{TB}(T_{D})}\left({\left|\frac{d{\tilde{L}}_{TB}}{dT}\right|}_{T=T_{D}}+\frac{1}{pf(T_{D})}\left(A(L)+e^{rT}\frac{d}{dT}\left(\overset{\;T}{\underset{\;0}{\int }}\;S({\tilde{L}}_{NTB}(t))e^{-rt}\;dt\right)\right)\right). \end{array}$$

Unfortunately, due to the complexity of Eq. (12), we are unable to deduce the absolute effect on the optimal rotation length and are thus restricted to using numerical analysis. However, for a special case, when the non-timber benefits are not affected by disease, the first-order condition can be found since ${\tilde{L}}_{NTB}(T)=L$ as *σ*_*i*_ = 1∀*i* in Eq. (10). Under this restriction the first-order condition is

(13) $${\left(\frac{1}{f(T_{D})}\frac{df(T)}{dT}\right|}_{T=T_{D}}-r=\frac{1}{{\tilde{L}}_{TB}(T_{D})}\left({\left|\frac{d{\tilde{L}}_{TB}}{dT}\right|}_{T=T_{D}}+\frac{A(L)-S(L)}{pf(T_{D})}\right).$$

Eq. (13) shows that when disease does not reduce the non-timber benefits, the optimal rotation length (*T* = *T*_*D*_) is obtained when the relative marginal benefit of waiting for one more instant of timber growth minus the discount rate (left-hand side) is equal to the relative marginal loss of the disease spreading further, and the future land rent minus the benefit of accruing the green payment relative to the timber benefit (right-hand side). We know that the inclusion of disease can have a mixed effect on the optimal rotation length due to the trade-off between waiting for the timber to grow and the cost of allowing infection to spread further over time (Macpherson et al., 2016). Eq. (13) shows that the inclusion of non-timber benefits (which remain unaffected by disease) will act to increase the optimal rotation length. However, without knowing the magnitude of the terms it is impossible to say what the net outcome will be compared with the disease-free case.

## 3 A Numerical Model

### 3.1 Timber Volume Function

**Fig. 1:**
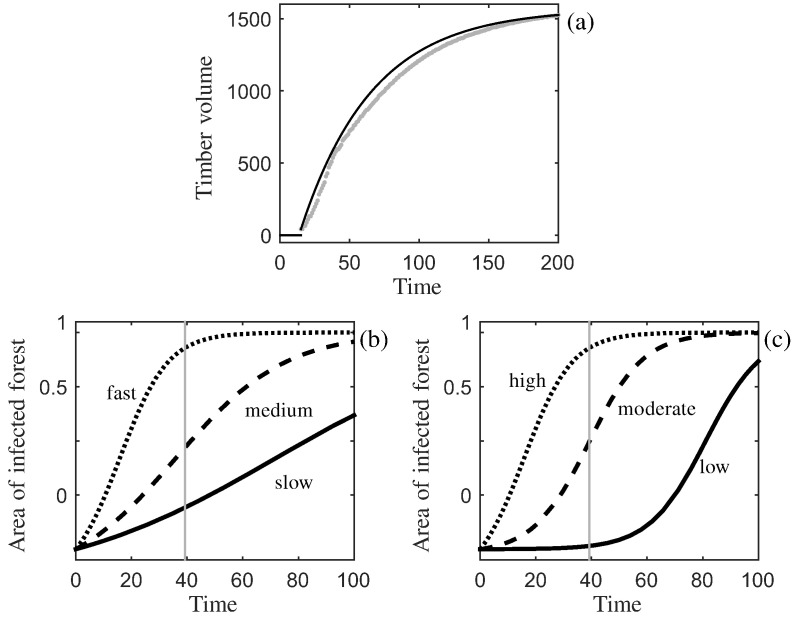
Timber volume and disease progress curves. In (a) the data points (grey dots) are the timber volume (m^3^ ha^ −1^) from the Forest Yield model for unthinned, yield class 14 *Picea sitchensis* against time (years). The fitted curve (black) is produced using Eq. (14) and the parameters are in Table 1. The area of infected forest (*L* − *x*(*t*) ha) against time (years) is plotted with (b) a fixed rate of primary infection and three secondary infection rates and (c) a fixed rate of secondary infection and three primary infection rates (the parameter sets are in Table 2). The optimal rotation length of the disease-free system, *T*_*DF*_, is shown as a vertical, grey line.

In our framework the net benefit at the end of the rotation is dependent on the function describing how the volume of timber grows over time, *f*(*T*). In this paper we use the example of a yield class of 14 (growth in timber volume of approximately 14  cubic metres per hectare per year), as typical of the growth rate of *Picea sitchensis* (sitka spruce). Sitka spruce is the dominant species used for timber production in Scotland and elsewhere in the British uplands (Forestry Commission, 2011) because it is fast growing and well suited to moist and well-drained soils. The model “Forest Yield” developed by the government agency Forest Research was used to estimate the average timber volume per tree and density of trees (number per hectare) over time (Matthews et al., 2016), which allowed us to estimate the average timber volume per hectare. These data points are shown in Fig. 1 (a) where the timber volume of a hectare of forest (*V*_*i*_) is given for each time step (*T*_*i*_). (*T*_1_,*V*_1_) is the point recorded once the average tree has grown into the 7–10  cm range of diameter at breast height (DBH); trees are generally not commercially harvested at smaller sizes. This model includes the natural mortality rate that is expected of an un-thinned stand with 2 m initial tree spacing.

Using the model output we can fit a curve which has the form

(14) $$f(T)=\left\{\begin{array}{ll} 0\; & \text{if}\;T<T_{1}\\ V_{M}\left(1-e^{\bar{b}(T-T_{1})}\right)+V_{1}\; & \text{if}\;T\geq T_{1} \end{array}\right)$$

where (*T*_*M*_,*V*_*M*_) is the data point at the end of the time horizon. We used the growth model to obtain 185  years of output, and in order to capture the shape of the curve over time we fit parameter $\bar{b}$ by setting *f*(200) = *V*_*M*_. Moreover, since we are examining the effect of disease on the optimal rotation length, we include here the full time horizon output. All parameter values are given in Table 1, and Fig. 1 (a) shows the data points and fitted curve given by Eq. (14). Since trees are generally only harvested after they have reached 7–10  cm DBH, our model uses *T*_1_ as a lower harvesting boundary, where the trees will not be harvested before this time point.

### 3.2 Susceptible-Infected Compartmental Model

We now reduce the *N*-state compartmental model to a two-state, Susceptible-Infected (SI) system with *x*(*T*) representing the area of the susceptible forest and *y*(*T*) the area of the infected forest at time *T*. The total area of forest remains constant over time (*L* = *x*(*T*) + *y*(*T*)), therefore the SI system can be written as

(15a) $$\frac{dx}{dT}=-\beta x(T)\left(y(T)+P\right)$$

(15b) $$\frac{dy}{dT}=\beta x(T)\left(y(T)+P\right),$$

where the primary infection rate, *P*, controls the external infection pressure (e.g. from spores dispersed into the forest from some external source), and the secondary infection rate, *β*, controls the spread of infection within the forest (from infected to susceptible trees). Since the area of forest is constant (*dL*/*dT* = *dx*/*dT* + *dy*/*dT* = 0) we eliminate Eq. (15b) by setting *y*(*T*) = *L* − *x*(*T*). Thus the system reduces to

(16) $$\frac{dx}{dT}=-\beta x(T)\left(L-x(T)+P\right)$$

which can be solved using the separation of variables method to give

(17) $$x(T)=\frac{L+P}{\frac{P}{L}e^{(L+P)\beta T}+1}.$$

In the general framework, ${\tilde{L}}_{TB}(T)$ and ${\tilde{L}}_{NTB}(T)$ represent the effective area of the forest providing the timber and non-timber benefit respectively (Eqs. (8) and (10)). For the SI system we have

(18) $${\tilde{L}}_{TB}(T)=x(T)+\rho (L-x(T))$$

and

(19) $${\tilde{L}}_{NTB}(T)=x(T)+\sigma (L-x(T))$$

where *ρ* scales the timber revenue from infected trees, and *σ* scales the green payment from infected trees. Both 0 ≤ *ρ* ≤ 1 and 0 ≤ *σ* ≤ 1 hold, and setting *ρ* = 1 (or *σ* = 1) means that the infection has no effect on the timber (or non-timber) benefit from infected trees; conversely *ρ* = 0 (or *σ* = 0) means that there is no timber (or non-timber) benefit from infected trees.

The dynamics in Eq. (17) are governed by the primary and secondary infection rates. We select six parameter sets (detailed in Table 2) with the aim of capturing the characteristics of different diseases caused by different pathogen species. The rate of disease progress (change in area of infected forest over time) is shown in Fig. 1 (b) and (c). It may be possible to estimate the secondary infection rate from epidemiological field data, however interpreting and quantifying an appropriate rate of primary infection is more difficult. We therefore introduce another parameter *t*_0.5_, which is the time taken for half the forest to become infected, to describe the primary infection rate (for a fixed secondary infection rate). Using Eq. (17) we can find this value by setting *x*(*t*_0.5_) = 0.5*L* giving

(20) $$t_{0.5}=\frac{\ln(L/P+2)}{(L+P)\beta }.$$

We can equate *t*_0.5_ to the disease-free rotation length, or proportions of it, to enable an easy interpretation of the effect of variation in the primary infection rate (when the secondary infection rate is fixed). For example, *t*_0.5_ = *T*_*DF*_ corresponds to half of the trees in the forest being infected by the end of a disease-free rotation length. Fig. 1 (b) and (c) shows disease progress curves generated for the parameter sets in Table 2. (Note that we also give *t*_0.5_ for the first set of parameters when *P* is constant and *β* is fixed – this was done in order to find appropriate levels of *β*.)

## 4 General Results

In this section we set the land rent after harvest, *a*, to zero and use the timber volume function and compartmental disease model defined in Section 3 to give further insight into the results found in Section 2.

### 4.1 No Disease

First we analyse the system without disease to provide baseline results that can be used to measure the effect of disease on the system. Recalling that the optimal rotation length is given by the first-order condition in Eq. (4), we now substitute the timber volume function in Eq. (14) to obtain

(21) $$-\frac{\bar{b}V_{M}e^{\bar{b}(T-T_{1})}}{V_{M}(1-e^{\bar{b}(T-T_{1})})+V_{1}}-r=-\frac{s}{p(V_{M}(1-e^{\bar{b}(T-T_{1})})+V_{1})}.$$

**Fig. 2:**
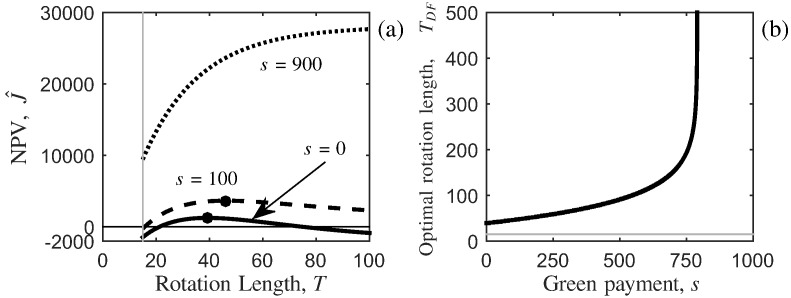
The effect of a green payment for non-timber benefits on the optimal forest rotation length for the system without disease. In (a) the NPV in Eq. (1) is plotted against the rotation length *T* (years) for three levels of green payment (*£* ha^ −1^ year^ −1^): *s* = 0 (solid black), *s* = 100 (dashed black) and *s* = 900 (dotted black). A black circle marks the optimal rotation length that maximises the NPV for the first two cases (for the third case the optimal rotation length is infinite). In (b) the variation in the optimal rotation length, *T*_*DF*_ (years), in Eq. (22) is plotted against the green payment, *s* (*£* ha^ −1^ year^ −1^). Note that when the green payment is greater than *s*^(*∞*)^ = 790.31 (Eq. (23) the optimal rotation length becomes infinite. In all plotted relationships the growth function is parameterised for yield class 14 *Picea sitchensis* where the minimum harvesting boundary, *T*_1_, is given by the vertical grey line in (a) and horizontal grey line in (b). The land rent is set to zero, and all other parameters can be found in Table 1.

Solving for the optimal rotation length (*T* = *T*_*DF*_) we have

(22) $$T_{DF}=\frac{1}{\bar{b}}\ln\left(\frac{s-pr(V_{M}+V_{1})}{pV_{M}(\bar{b}-r)}\right)+T_{1},$$

which exists when *s* < *pr*(*V*_*M*_ + *V*_1_), since $\bar{b}<0$. Let

(23) $$s^{\left(\infty \right)}=pr(V_{M}+V_{1})$$

be the level of green payment where the optimal rotation length becomes infinite. When *s* < *s*^(*∞*)^, the optimal rotation length is where the maximum NPV is achieved (black dot on the dashed and solid curves in Fig. 2 (a)); that is where waiting for one more instant of timber growth and non-timber benefits (through the green payment) is equal to the opportunities forgone (the profit that could be obtained from investing elsewhere, such as a bank). However, when *s* ≥ *s*^(*∞*)^, then the optimal rotation length will be infinite (dotted curve in Fig. 2 (a)). The green payment is designed so that there is a benefit from retaining the cover of the current tree crop unharvested for longer, and this is seen further in Fig. 2 (b) where, as *s* → *s*^(*∞*)^, then *T*_*DF*_ →*∞*, and the optimal rotation length becomes infinite. This results in the optimal harvesting strategy changing from clear-felling to permanently retaining tree cover, thus turning the forest into an amenity forest (producing only non-timber benefits). However, the result of an infinite optimal rotation length is likely to be due to setting the future land rent to zero (or the omission of multiple rotations), thus removing the benefit after the first rotation. Increasing the price of timber, *p*, or the discount rate, *r*, will increase the level of green payment needed for the optimal rotation length to become infinite (Eq. (23)), since this will increase the benefit of acting sooner (by increasing the value of the timber and decreasing the future benefits respectively).

### 4.2 Disease

We now find the optimal rotation length for the system with disease, *T* = *T*_*D*_, which maximises the NPV in Eq. (6) when the forest volume function is of the form of Eq. (14), and the disease follows the SI compartmental model, with the area of susceptible forest over time given by Eq. (17). We first assume that the non-timber benefit remains unaffected by disease (Section 4.2.1), and then relax this restriction so that the disease affects both timber and non-timber benefits (Section 4.2.2).

#### 4.2.1 The Optimal Rotation Length when Disease Affects the Timber Benefit Only

When the green payment remains unaffected by disease, Eq. (10) reduces to ${\tilde{L}}_{NTB}(T)=L$. An analytical solution for the optimal rotation length is intractable, therefore we carry out analysis of sensitivity to the parameters controlling the spread of infection (*β* and *P*), and the revenue from timber of infected trees (*ρ*).

First, setting *ρ* = 0 simplifies the model as the net benefit of the timber at the end of the rotation is dependent on the area of healthy forest only, that is ${\tilde{L}}_{TB}(T)=x(T)$ from Eq. (18). Substituting this and the timber volume function (Eq. (14)) into the first-order condition in Eq. (13), we find

(24a) $$\frac{1}{f(T)}\frac{df}{dT}-r=\frac{1}{x(T)}\left|\frac{dx}{dT}\right|-\frac{S(L)}{pf(T)x(T)}$$

(24b) $$\begin{array}{ll} \;⟹\; & \frac{-V_{M}\bar{b}e^{\bar{b}(T-T_{1})}}{V_{M}(1-e^{\bar{b}(T-T_{1})})+V_{1}}-r=\frac{P\beta (L+P)}{P+Le^{-(L+P)\beta T}}\\ & -\frac{s(Pe^{(L+P)\beta T}+L)}{p(L+P)(V_{M}(1-e^{\bar{b}(T-T_{1})})+V_{1})}. \end{array}$$

**Fig. 3:**
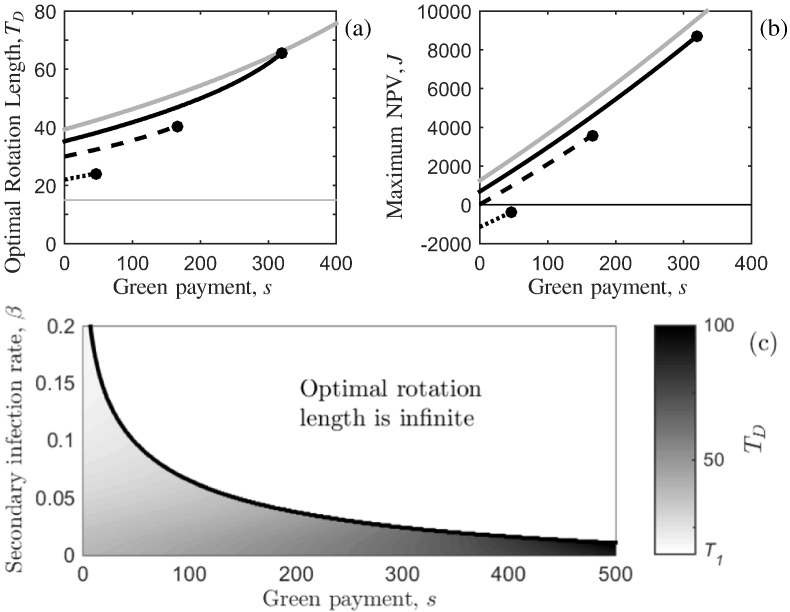
The effect of varying the secondary infection rate on the optimal rotation length when disease affects the timber benefit only. Variation in (a) the optimal rotation length, *T*_*D*_, and (b) the maximum NPV in Eq. (6) with the level of green payment *s* (*£* ha^ −1^ year^ −1^) when the timber that is infected is worth nothing (*ρ* = 0). Three rates of secondary infection, *β*, are shown: slow (solid black), medium (dashed black) and fast (dotted black), with parameter values as defined in Table 2. The system without disease in Eq. (1) is shown for comparison (grey). The black circles indicate the green payment value, $s_{D}^{\left(\infty \right)}$ where the optimal rotation length becomes infinite. This analysis is extended in (c) where the optimal rotation length is shown in a *s* − *β* (green payment – secondary infection rate) parameter space. The black curve is the boundary where the optimal rotation length becomes infinite: to the right of the black curve, *T*_*D*_ is infinite (represented by the white area and text stating so); and to the left of the black curve, *T*_*D*_ is finite and shown by a gradation in black-white shading with the grey-scale on the right-hand side indicating the optimal rotation length (where *T*_*D*_ = *T*_1_ is white and *T*_*D*_ = 100 is black). The rate of primary infection is set at the baseline value in all plots and other parameters can be found in Table 1.

It is clear that the green payment, *s*, has a positive effect on the optimal rotation length and maximum NPV (Fig. 3 (a) and (b)). However disease reduces the optimal rotation length and the maximum NPV (e.g. for each value of green payment in Fig. 3 (a) the optimal rotation length, when it exists, decreases as the rate of secondary infection, *β*, increases). We note that despite harvesting at the optimal time, the maximum NPV can be negative (this is true for the fast secondary infection rate in Fig. 3 (b)). A key point illustrated in Fig. 3 (a) is that, as in the disease-free case, once a critical value of green payment is realised (say at $s_{D}^{\left(\infty \right)}$, identified by the circles), it becomes optimal to never harvest the forest. This occurs for the following reason. Without a green payment the (negative) NPV is initially equal to the establishment costs. As time passes the trees grow and the present value of revenue from selling the timber increases, however the timber volume growth eventually saturates (Fig. 1 (a)), and thus the NPV reaches a maximum. If the trees are not harvested, the timber revenue will then decrease as *T* →*∞* (due to a decline in timber growth rate, discounting and disease), and the NPV tends to the establishment costs, *W*(*L*). Thus there is always one global stationary point in time which maximises the NPV (the “optimal rotation length”). The inclusion of a green payment, however, adds additional revenue (independent of tree growth and infection status) for as long as the trees remain unharvested, and we find that as *T* →*∞* then Ĵ→S(L)/r−W(L). Therefore, when the green payment is large enough, *S*(*L*)/*r* − *W*(*L*) will be greater than the value obtained at any other point in the rotation and thus it is optimal to retain tree cover and not to harvest.

Further analysis in Fig. 3 (c) shows the trade-off between waiting for the timber to grow, while accruing another instalment of the green payment, and the infection spreading further (and reducing the timber benefit) over time. The parameter space is split in two by a black curve representing where $s=s_{D}^{\left(\infty \right)}$: to the right, *T*_*D*_ is infinite, and to the left, *T*_*D*_ is finite. As before, Fig. 3 (c) highlights that increasing the green payment (which is not dependent on the level of disease) leads to increases in the optimal rotation length which, once a critical level of green payment is reached, becomes infinite; when the rate of secondary infection is increased, a smaller level of green payment is required for the optimal rotation length to become infinite. This occurs since disease reduces the revenue from the harvested timber and thus decreases the benefit of delaying harvest. Note that on the x-axis of Fig. 3 (c), *β* = 0 and so the system simplifies to the disease-free case and the optimal rotation length (for the system with disease), when it exists, will not be greater than the system without disease; moreover the parameter space where *T*_*D*_ = *∞* will meet the x-axis at $s_{D}^{\left(\infty \right)}=s^{\left(\infty \right)}$ (as would be seen if the x-axis range was extended).

It is possible that timber from infected trees can still generate some revenue. Using the same method as before, we carry out analysis of sensitivity to parameter *ρ* by substituting the function describing the effective area of forest providing timber benefits, ${\tilde{L}}_{TB}(T)=x(T)(1-\rho )+\rho L$, and timber volume function (Eq. (14)) into the first order condition (Eq. (13)) and get

(25a) $$\frac{1}{f(T)}\frac{df}{dT}-r=\frac{1}{{\tilde{L}}_{TB}(T)}\left(\left|\frac{d{\tilde{L}}_{TB}(T)}{dT}\right|-\frac{S(L)}{pf(T)}\right)$$

(25b) $$\begin{array}{ll} \;⟹\; & \frac{-V_{M}\bar{b}e^{\bar{b}(T-T_{1})}}{V_{M}(1-e^{\bar{b}(T-T_{1})})+V_{1}}-r=\frac{Pe^{(L+P)\beta T}+L}{L+P\left(1+\rho (e^{(L+P)\beta T}-1)\right)}\\ & \times \left(\frac{\beta P{\left(L+P\right)}^{2}e^{(L+P)\beta T}(1-\rho )}{{\left(Pe^{(L+P)\beta T}+L\right)}^{2}}-\frac{s}{p(V_{M}(1-e^{\bar{b}(T-T_{1})})+V_{1})}\right). \end{array}$$

**Fig. 4:**
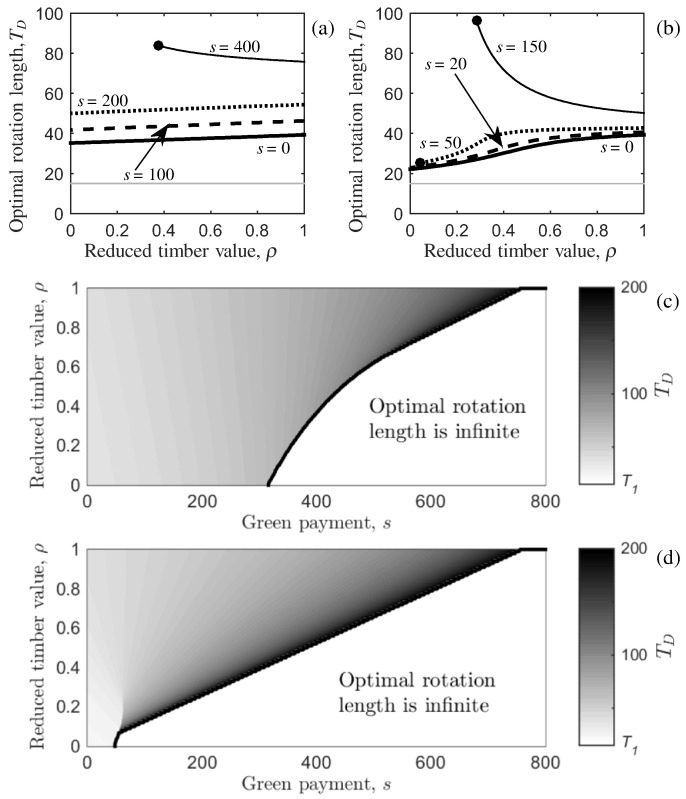
The effect of varying the reduction in timber value caused by disease on the optimal rotation length when disease affects the timber benefit only. Variation in the optimal rotation length, *T*_*D*_, with the reduction in timber value caused by disease, *ρ*, for (a) slow and (b) fast rates of secondary infection. The value of the green payment, *s* (*£* ha^ −1^ year^ −1^) is given next to each curve. The horizontal, grey line represents the lower harvesting boundary, *T*_1_. The optimal rotation length, *T*_*D*_, is shown in a *s* − *ρ* parameter space for (c) slow and (d) fast rates of secondary infection. The black curves represent the boundary where the optimal rotation length becomes infinite: to the right of the black curves, *T*_*D*_ is infinite (represented by the white area and text stating so); and to the left of the black curve, *T*_*D*_ is finite and shown by a gradation in black-white shading with the grey-scale on the right-hand side indicating the optimal rotation length. The primary infection rate is at the baseline in all plots (Table 2), and all other parameters are given in Table 1.

When *ρ* = 1, Eq. (25b) reduces to the disease-free system and the optimal rotation length is given by Eq. (22). Interestingly, decreasing the value of timber from infected trees can result in either an increase or a decrease in the optimal rotation length dependent on the level of green payment and how fast the infection spreads (Fig. 4 (a) and (b)). For example, when the secondary infection rate is slow, as *ρ* is decreased from 1 to 0 the optimal rotation length decreases when *s* ≤ 200, but increases when *s* = 400 (Fig. 4 (a)). When the secondary infection rate is fast the behaviour is the same, although a smaller green payment is required for the optimal rotation length to increase (e.g. a payment of *s* = 150 is shown to be sufficient in Fig. 4 (b)). This key result is highlighted further in Figs. 4 (c) and (d) where the optimal rotation length is shown in a *s* − *ρ* parameter space for slow and fast secondary infection rates respectively: as *ρ* is decreased, the optimal rotation length will change depending on whether *s* is less than or greater than $s_{D}^{\left(\infty \right)}$ (the green payment required for the optimal rotation length becomes infinite when *ρ* = 0). When the green payment is less than $s_{D}^{\left(\infty \right)}$ the optimal rotation length will decrease as *ρ* is decreased. Alternatively, when the green payment is greater than $s_{D}^{\left(\infty \right)}$, the optimal rotation length will increase as *ρ* is decreased and eventually become infinite (the white region of the parameter space to the right of the black curve in Fig. 4 (c) and (d)).

Fig. 4 highlights the complex interaction between the rate of secondary infection, the effect of disease on timber value, and the green payment. The decline in the optimal rotation length as the timber value of infected trees decreases is easily understood, because the NPV is reduced thus motivating an earlier harvest to increase the proportion of timber that comes from uninfected trees. The increase in the optimal rotation length when $s>s_{D}^{\left(\infty \right)}$ can be understood as the non-timber benefit, which is dependent on the retention of unharvested trees, outweighing the timber benefit. When the infection spreads quickly (Fig. 4 (b) and (d)), most of the forest is infected by the time the trees have grown above the minimum tree-size harvesting boundary, and thus a majority of the timber is subject to the reduced value. Therefore, there is a benefit in letting the trees grow larger before harvest and accruing the green payment for non-timber benefits for longer. When the infection spreads slowly (Fig. 4 (a) and (c)) the effect of the disease on the timber benefit is less, thus a greater annual green payment value is required to motivate delaying harvest.

We have carried out a similar analysis of sensitivity to the primary infection rate, *P*, of Eq. (6), and it showed that increasing *P* had a similar effect on the optimal rotation length as increasing the secondary infection rate, *β*. More specifically, a disease which arrives early (high *P*) and transmits slowly (small *β*) has a similar effect on the optimal rotation length to a disease which arrives late (low *P*) and transmits fast (big *β*).

#### 4.2.2 The Optimal Rotation Length when Disease Affects the Timber and Non-timber Benefit

**Fig. 5:**
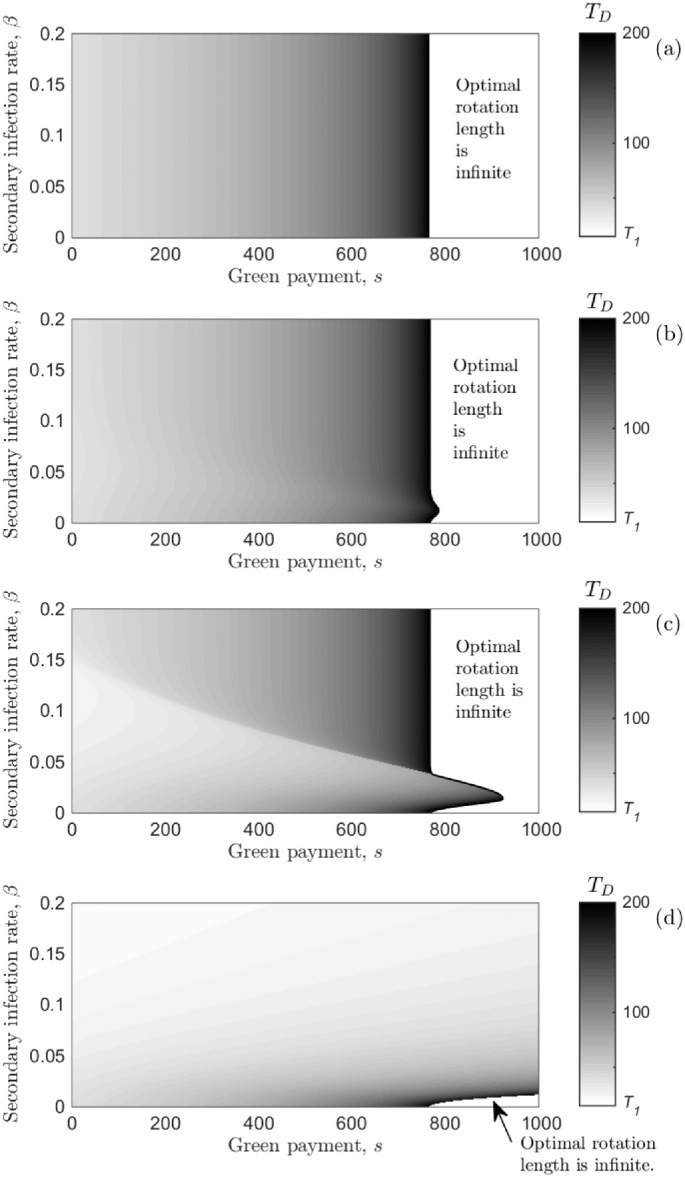
The effect of varying the reduction in timber and non-timber benefits caused by disease. The optimal rotation length, *T*_*D*_, is shown against the green payment, *s* (*£* ha^ −1^ year^ −1^), and secondary infection rate, *β*, when timber (*ρ*) and non-timber (*σ*) benefits are reduced by disease: (a) *ρ* = *σ* = 1, (b) *ρ* = *σ* = 0.8, (c) *ρ* = *σ* = 0.2 and (d) *ρ* = *σ* = 0. The black boundary indicates where the optimal rotation length becomes infinite, $s_{D}^{\left(\infty \right)}$: to the right, *T*_*D*_ is infinite (represented by the white area and text stating so); and to the left, *T*_*D*_ is finite and the gradation in black-white shading gives the optimal rotation length, which is identified by the grey-scale on the right-hand side of the plots. For the disease-free system, the level of green payment required for the optimal rotation length to become infinite is *s*^(*∞*)^ ≈ 790 (*β* = 0). The primary infection rate is at the baseline in all plots (Table 2), and all other parameters are given in Table 1.

We now investigate what happens when the timber *and* non-timber benefits are dependent on the infection state of the forest, as given by $S({\tilde{L}}_{NTB}(T))=s{\tilde{L}}_{NTB}(T)$ (Eq. (9b)) and the first-order condition in Eq. (12). To simplify the problem, we set *σ* = *ρ* meaning that the disease reduces the timber benefit from infected trees and non-timber benefit from infected trees equally, and use numerical optimisation to find how the optimal rotation length varies with changes in the level of green payment, *s*, and rate of secondary infection, *β*, in Fig. 5 for four levels of reduction in timber and non-timber benefits (due to disease).

First, when disease does not affect the timber and non-timber benefits (*ρ* = *σ* = 1) the optimal rotation length is the same as the disease-free case in Eq. (22). As the level of green payment, *s*, is increased, the optimal rotation length will increase and eventually become infinite at *s* = *s*^*∞*^ (Fig. 5 (a) and also Fig. 2 (b)). Decreasing the value of timber and non-timber benefits from infected trees (decreasing *σ* and *ρ* equally) creates a key trade-off between waiting for the timber to grow, while accruing another instalment of the green payment, and the infection spreading further (reducing both the timber and non-timber benefits). Consider the parameter space where *s* < *s*^(*∞*)^ in Fig. 5 (b) and (c) (where *s*^(*∞*)^ is the level of green payment needed for the optimal rotation length to become infinite when *β* = 0 and thus there is no disease). Taking a vertical transect for a fixed level of green payment shows that the optimal rotation length initially decreases as the rate of secondary infection, *β*, increases, but once a critical value of *β* is reached, the optimal rotation length starts to increase. Initially, there is an economic benefit from decreasing the optimal rotation length and salvaging uninfected timber due to the slow rate of secondary infection. However, once the secondary infection rate is increased sufficiently, the economic benefit from waiting for further tree growth and accruing another instalment of the green payment is increased, since the proportion of infected trees in the forest will not substantially increase in the following years (due to a large fraction of the forest already being infected). As the level of green payment is increased (but is still less than *s*^(*∞*)^), the optimal rotation length starts to increase for smaller values of *β* (e.g. in Fig. 5 (c), when *s* = 200, the optimal rotation length decreases as *β* is increased from 0 to 0.105, and increasing *β* further increases the optimal rotation length; whereas when *s* = 600, the change from a decrease to an increase in the optimal rotation length occurs at *β* = 0.052). A key result is therefore that slower transmitting diseases require a greater level of green payment to incentivise retaining tree cover for longer. Moreover, we also note that the degree of variation in optimal rotation length (as *β* is increased) is sensitive to the level of reduction in the timber and non-timber benefits: when the reduction is small (*ρ* = *σ* = 0.8) there is little change in the optimal rotation length (Fig. 5 (b)); alternatively when the reduction is large (*ρ* = *σ* = 0.2), the optimal rotation length experiences large variation (as identified by the change in shade in the grey-scale in Fig. 5 (c)).

There exists a level of green payment, $s_{D}^{\left(\infty \right)}$, where it is optimal to never harvest the forest (to the right of the black boundary in Fig. 5 - the boundary shows $s_{D}^{\left(\infty \right)}$). This value is dependent on the secondary infection rate, *β*, and the reduction in the value of timber and non-timber benefits caused by disease. When the timber and non-timber benefit have a positive, but reduced, value (0 < *ρ* = *σ* < 1), for the majority of the *β* parameter range the optimal rotation length will become infinite at the same level of green payment as the disease-free case (e.g. $s_{D}^{\left(\infty \right)}=s^{\left(\infty \right)}\approx 790$). The exception is for a range of small (non-zero) values of *β* where the optimal rotation is finite compared with the disease-free system (which would be infinite), giving $s_{D}^{\left(\infty \right)}>s^{\left(\infty \right)}$. We can understand why this happens as follows. When the secondary infection rate is very small (*β* ≈ 0), disease has very little impact on both timber and non-timber benefits, thus the system is similar to the disease-free system where the optimal rotation length becomes infinite at $s_{D}^{\left(\infty \right)}=s^{\left(\infty \right)}$. Increasing *β*, reduces the timber and non-timber benefits, therefore there is an incentive to harvest the forest to salvage some (uninfected) timber, and thus a greater level of green payment is required for the optimal rotation length to become infinite (i.e. $s_{D}^{\left(\infty \right)}>s^{\left(\infty \right)}$, which is shown by the displacement to the right of the black boundary over a range of *β* in Fig. 5 (b) and (c)). Increasing *β* again results in a higher proportion of the forest being infected earlier in the rotation, thus the benefit of waiting is small and the value of green payment required for the optimal rotation length to become infinite reduces back to $s_{D}^{\left(\infty \right)}=s^{\left(\infty \right)}$.

When the infection spreads quickly with a high value of *β*, so that a high proportion of the forest is infected relatively soon after planting, then the NPV reduces to

(26) $$Ĵ(T)\to -cL+\rho pf(T)Le^{-rT}+\frac{\sigma sL}{r}(1-e^{-rT}),$$

since *β* →*∞* (and ${\tilde{L}}_{TB}(T)\to \rho L$ and ${\tilde{L}}_{NTB}(T)\to \sigma L$). We can find the optimal rotation length for when this is the case by differentiating Eq. (26) and setting it equal to zero giving

(27) $$T_{D}\to \frac{1}{\bar{b}}\log\left(\frac{\sigma s-\rho pr(V_{M}+V_{1})}{\rho pV_{M}(\bar{b}-r)}\right)+T_{1}.$$

Since *σ* = *ρ*, Eq. (27) means that the optimal rotation length will be the same as the disease-free system (Eq. (22)), and become infinite at the same level of green payment (e.g. when *s* = *pr*(*V*_*M*_ + *V*_1_)). This is shown in Fig. 5 (b) and (c) where the black boundary indicating $s_{D}^{\left(\infty \right)}$ is equal to *s*^(*∞*)^ for a wide range of values of *β* (note that when *β* ≥ 0.05, at least 99% of the forest will be infected 34  years after planting).

When infected trees provide no timber or non-timber benefits (*σ* = *ρ* = 0), increasing the rate of secondary infection, *β*, decreases the optimal rotation length across the range of levels of green payment, *s* (Fig. 5 (d)). Moreover, the level of green payment required for the optimal rotation length to become infinite is much higher compared with the case without disease (i.e. $s_{D}^{\left(\infty \right)}>s^{\left(\infty \right)}$), or the system with a fast transmitting disease and *σ* = *ρ* > 0. This happens because there is a greater incentive to salvage harvest (uninfected) timber and forgo the non-timber benefits (which are declining with the spread of infection), which is unlike the previous case (where infected trees still provided timber and non-timber benefits, albeit reduced by disease).

We have carried out a similar analysis of sensitivity to the primary infection rate, *P*, of Eq. (6), and showed that when both the timber and non-timber benefits are reduced by disease, increasing *P* had a similar effect on the optimal rotation length to that of increasing the secondary infection rate, *β*. More specifically, the optimal rotation length became infinite in the disease-free case for a wide range of *P* values. The exception to this was when the reduction in timber and non-timber benefits was large, then for a small range of *P* a larger green payment was required for the optimal rotation length to become infinite.

## 5 Summary and Discussion

The interaction between the effects of a green payment rewarding land managers for the non-market benefits provided by their forests and tree disease characteristics is the central issue for this paper. Where a disease arrives during a forest rotation the optimal rotation length, which maximises the NPV of the forest, is found when the marginal benefit of waiting for one more instant of timber growth and accruing of green payment, is equal to the value of the opportunities forgone and the marginal cost of the disease spreading further (Section 2). In Macpherson et al. (2016) we showed that when disease reduces the value of timber from infected trees, the optimal rotation length of a plantation forest is generally decreased if the infection spreads slowly, but delayed to the disease-free optimal rotation length if the infection spreads quickly. In this paper, we further show that the inclusion of a green payment based on the retention of trees counteracts the effect of disease on shortening the optimal rotation length, since the green payment incentivises the owner to delay harvesting. Analysis of sensitivity to the parameters controlling the primary and secondary infection rate, and to the reduction in timber and non-timber benefits of infected trees, revealed that a complex trade-off arises between waiting for the trees to grow larger, accruing one more instalment of green payment, and the infection spreading further over time. Moreover, at some critical level of green payment, the optimal rotation length becomes infinite. When the pathogen reduces only the timber value, increasing the rate of primary and/or secondary infection reduces the level of green payment needed to generate an infinite optimal rotation length. However, when the disease affects both the timber and non-timber benefits equally, then the level of green payment required is the same as the disease-free system (with the exception of a narrow range of small values of the primary and/or secondary infection rates, where the level of green payment required for an infinite rotation length can be greater than the level required for the disease-free case). It has been shown previously that the inclusion of non-timber benefits (in the absence of disease) increases the optimal rotation length (Amacher et al., 2009), and interestingly in some cases the effect of forest carbon payments has been shown to increase the optimal rotation length and make it optimal never to harvest (Price and Willis, 2011, Van Kooten et al., 1995). However, one should view our result of infinite optimal rotation lengths with caution, since this could result from carrying out the analysis with the future land rent set to zero or because we do not consider multiple rotations. Another possible reason for obtaining an infinite optimal rotation length may be that we assume the green payment function to be linearly dependent on the forest area. This omits any saturation in the green payment that would be obtained if it was dependent on, say, the volume of timber.

The effect of catastrophic, abiotic events on forest owners' decision-making has been examined in several papers (Amacher et al., 2005, Amacher et al., 2009, Englin et al., 2000, Reed, 1984). Englin et al. (2000) carried out an empirical study using a Faustmann framework to find the effect of fire risk on a *Pinus banksiana* forest in the Canadian Shield region. They included the value of non-timber benefits (obtained through wilderness recreation), and found that while the presence of fire risk shortened the optimal rotation length, the inclusion of the non-timber benefits increased it. While we have a different model formulation (our framework is deterministic rather than stochastic), our overall results have notable similarities with those of Englin et al. (2000), except for our finding of parameter spaces where the optimal rotation length becomes infinite. Moreover, this is particularly interesting since there are several dissimilarities between the effect of fire and disease on a forest system (which we listed in Section 1).

Our findings are important because they demonstrate the complex interaction between the effects of timber and non-timber values of a forest in the presence of tree disease. However, we have excluded many complexities in order to examine this interaction clearly. The most prominent of these is the omission of multiple rotations. As mentioned in Section 1, most optimal rotation length research considers multiple rotations where trees are perpetually planted and harvested, thus synonymously including the future stream of opportunity costs from using land for forestry (Amacher et al., 2009). Application of the traditional form of multiple rotation analysis (calculating the NPV over infinite forest rotations) to our study would require detailed knowledge of the persistence of disease between rotations. For example, can the infection pressure change between rotations? In the absence of such knowledge, with modeling therefore restricted to a single rotation, a simple way to include the value of the land after the first tree crop is harvested would be to include a future land rent term where a net payment is received annually after the harvest of the first rotation. This can represent changing the land use or changing the tree species planted in the following rotation, which may be necessary after an epidemic that retains infectious material within the site (as is the case for *Heterobasidion annosum*; Pratt, 2001, Redfern et al., 2010). Therefore, we included a future land rent term in the model framework (Eq. (12)), but set the annual payment to zero when carrying out our study so as to concentrate on the effect of the disease within one rotation of a multiple-output forest. The first-order condition showed that the effect of disease on the optimal rotation length is dependent on the future land rent minus the green payment that is dependent on the retention of unharvested trees, and so the addition of future land rent would counteract the effect of the green payment. Thus, post-harvest land rent incentivises harvesting earlier and increases the level of green payment needed to switch to an amenity forest with the current tree crop (infinite optimal rotation length). Our decision (for this reason) to omit land rent in this study, may explain why we found that the optimal rotation length becomes infinite for certain ranges of green payment values, whereas Englin et al. (2000) did not.

In this paper, we consider the case when disease reduces the timber and non-timber benefits in equal proportions. However, our model could be used to examine the effect of an unequal reduction by disease in the timber and non-timber benefits on the optimal rotation length. Although we have not carried out the analysis here, Eq. (27) shows that for an infection which spreads quickly, if the reduction in the non-timber benefit is greater than the reduction in timber benefit then the level of green payment needed for an infinite optimal rotation length would increase (and decrease if the effect of disease on the non-timber benefit was smaller than its effect on the timber benefit). Moreover, the green payment function subsumes all non-timber benefits into one term. We assume that this term is linearly dependent on the area of the tree cover which may be representative of certain non-timber benefits (such as recreation). However, many non-timber benefits may depend on other forest attributes such as the age of the trees or their biomass (which is linked to the volume of timber). To include this in our model, the green payment function can be modified so that each non-timber benefit (or at least the ones which are being investigated), is represented separately within the overall model framework. This would mean that each ecosystem service would have its own green payment term dependent on the appropriate forest attribute(s). For example, a green payment for biodiversity may depend on the age of the forest, whereas a green payment for carbon sequestration may depend on the timber volume.

Moreover, this set-up allows the effect of disease on the range of non-timber benefits to be included individually, which may be important if the disease affects ecosystem services differently. For example, the dominant effect of a disease like *D. septosporum* may only be to reduce timber volume: this would affect the timber benefit as well as the carbon sequestration; but the recreational and biodiversity benefits may remain largely unaffected. This framework should facilitate a better understanding of the trade-offs between the ecosystem services delivered by forests and how disease would affect them when considering the optimal time to harvest a particular host-pathogen forest system. However, in practice, it may be difficult to construct this framework because of the difficulty in characterising the specific details of the ecosystem services and the effect of disease on them, e.g. there are many metrics which evaluate the level of ‘biodiversity’ in a forest (Ferris and Humphrey, 1999).

The key results in this paper also have implications for the likelihood of adoption of forest management options that influence the spread of infection. The inclusion of a green payment increases the optimal rotation length and thus the period of time that infected trees are left standing and potentially acting as a reservoir of infection that can spread to surrounding forests. It also increases the exposure time of these unhealthy (or even dead) trees to further disturbances such as fire, wind, pests and other pathogens (Spittlehouse and Stewart, 2003). Never harvesting an infected forest would be seen by many as irresponsible, especially for fast-spreading epidemics, and is contrary to government prescriptions for combating some diseases such as *P. ramorum* in Great Britain (Forestry Commission Scotland, 2015). This highlights the importance of scaling, and we are cautious about extrapolating a strategy which was optimised from a single forest owner's perspective to a landscape or even regional scale. To do this would require a different model framework, since the decision to harvest in each forest affects the risk of disease transmission to neighbouring forests. One such framework is to use a network model (Keeling and Eames, 2005), where the nodes in the network represent forest patches, owned by different managers, who are connected dependent on how the infection spreads. Each forest manager decides when to harvest their patch by maximising their forest patch's NPV (a myopic strategy). Thus, the decision of when to harvest a patch will be (indirectly) dependent not only on the patch's infection status, but also on the connected neighbouring patches' infection status and harvesting decisions. We expect that an increase in the optimal rotation length (and a switch to an infinite optimal rotation length) would be less apparent within this type of model since there is now an additional cost of allowing the disease to spread. This would facilitate a better understanding of the effect of disease spread on the optimal rotation length at a landscape scale.

In summary, we extend the generalisable bioeconomic model in Macpherson et al. (2016), which combines the Faustmann model with an epidemiological compartmental model, to find the effect of disease on the optimal rotation length of a multiple-output forest. We show that a payment for the non-timber benefits will act to increase the optimal rotation length. However, we also found a complex trade-off with the disease characteristics. This framework can be easily extended to examine a specific host-pathogen system, or to investigate the trade-offs between ecosystem services and disease, and the effect that this has on the optimal rotation length.

## Figures and Tables

**Table 1:** Parameter definitions, baseline values and range of values tested in sensitivity analyses.

Parameter	Definition	Baseline value	Sensitivity range
*L*	Area of forest	*L* = 1 ha	–
*c*	Forest establishment cost^a^	*c* = £1920 ha^ −1^	–
*p*	Price of timber^b^	*p* = £16.79 m^ −3^	–
*s*	Annual green payment (*£* ha^ −1^)	–	*s* ∈ [0,1000]
*a*	Annual land rent after harvest	*£*0 ha^ −1^	–
*r*	Discount rate	*r* = 0.03	–
*f*(*T*)	Timber volume growth (m^3^ ha^ −1^)	Eq. (14)	–
(*T*_*i*_,*V*_*i*_)	Time and volume (years and m^3^ ha^ −1^)^c^	(*T*_1_,*V*_1_) = (15,43)	–
$\bar{b}$	Fitted parameter in *f*(*T*)	$\bar{b}=-0.01933$	–
${\tilde{L}}_{TB}(T)$	Effective area providing timber benefit	Eq. (8)	–
${\tilde{L}}_{NTB}(T)$	Effective area providing non-timber benefit	Eq. (10)	–
*β*	Secondary infection rate	Table 2	*β* ∈ [0,0.2]
*P*	Primary infection rate	Table 2	*P* = [0.0003,0.019,0.16]
*t*_0.5_	Time taken for the susceptible area to halve	Table 2	–
*ρ*	Timber revenue from infected trees	–	*ρ* ∈ [0,1]
*σ*	Non-timber benefit from infected trees	–	*σ* ∈ [0,1]

a The net cost of planting is taken to be zero on the basis that the gross cost is the same as the government subsidy payments available for Woodland Creation (in the form of an initial planting payment; https://www.ruralpayments.org/publicsite/futures/topics/all-schemes/forestry-grant-scheme/woodland-creation/).

b The price of timber is the average standing price (per cubic metre overbark) taken from the Coniferous Standing Sales Price Index for Great Britain on 19th May 2016 (http://www.forestry.gov.uk/forestry/INFD-7M2DJR).

c Parameters values are taken from the Forest Yield model of Forest Research in Great Britain for yield class 14 *Picea sitchensis* without thinning and with a 2-m initial spacing (2500 trees ha^ −1^).

**Table 2:** Parameter sets for the primary and secondary infection rates.

Disease dynamics (Primary – Secondary)	*P*	*β*	*t*_0.5_
High – Fast	0.16^B^	0.1^B^	*t*_0.5_ = *T*_*DF*_/2
High – Medium	0.16	0.044	*t*_0.5_ = *T*_*DF*_
High – Slow	0.16	0.022	*t*_0.5_ = 2*T*_*DF*_
High – Fast	0.16	0.1	*t*_0.5_ = *T*_*DF*_/2
Moderate – Fast	0.019	0.1	*t*_0.5_ = *T*_*DF*_
Low – Fast	0.0003	0.1	*t*_0.5_ = 2*T*_*DF*_

B Denotes the baseline value for the primary and secondary infection rate.
